# MYCN and KAT2A form a feedforward loop to drive an oncogenic transcriptional program in neuroblastoma

**DOI:** 10.1038/s41389-025-00557-2

**Published:** 2025-04-24

**Authors:** Zhihui Liu, Jason J. Hong, Xiyuan Zhang, Carly M. Sayers, Wendy Fang, Man Xu, Sydney Loria, Sakereh Maskal, Haiyan Lei, Haitao Wu, Rolf Swenson, Jordan L. Meier, Jack F. Shern, Carol J. Thiele

**Affiliations:** 1 Pediatric Oncology Branch, National Cancer Institute, Bethesda, MD USA; 2 Chemistry and Synthesis Center, National Heart, Lung, and Blood Institute, Bethesda, MD USA; 3 Chemical Biology Laboratory, National Cancer Institute, Frederick, MD USA

**Keywords:** Oncogenes, Embryonal neoplasms

## Abstract

The oncoprotein MYCN drives malignancy in various cancer types, including neuroblastoma (NB). However, our understanding of the mechanisms underlying its transcriptional activity and oncogenic function, as well as effective strategies to target it, remains limited. We discovered that MYCN interacts with the transcriptional coactivator KAT2A, and this interaction significantly contributes to MYCN’s activity in NB. Our genome-wide analyses indicate MYCN recruits KAT2A to bind to DNA, thereby transcriptionally regulating genes associated with ribosome biogenesis and RNA processing. Moreover, we identified that MYCN directly activates KAT2A transcription, while KAT2A acetylates MYCN, increasing MYCN protein stability. Consequently, MYCN and KAT2A establish a feedforward loop that effectively regulates global gene expression, governing the malignant NB phenotype. Treatment of NB cells with a KAT2A Proteolysis Targeting Chimera (PROTAC) degrader reduces MYCN protein levels, antagonizes MYCN-mediated gene transcription regulation and suppresses cell proliferation. This study highlights the potential of transcriptional cofactors as viable targets for developing anti-MYCN therapies.

## Introduction

*MYCN* belongs to the *MYC* family of oncogenic drivers, which includes c-Myc, L-Myc, and N-Myc [1–5]. First identified in neuroblastoma (NB), the *MYCN* gene encodes a basic helix-loop-helix-leucine zipper transcription factor (TF) named N-Myc or MYCN that is known to be dysregulated in a number of cancers including rhabdomyosarcoma, medulloblastoma, small cell lung cancer and prostate cancer [1, 2]. In NB, *MYCN* is a bona-fide oncogenic driver [1, 6–8] and it is known that the silencing of *MYCN* results in a decrease in cell proliferation and induction of cell differentiation in NB cells [9–11]. MYC oncoproteins play a critical role in initiating tumorigenesis, and their sustained activation is necessary for maintaining the neoplastic state in most human cancers, making them highly sought-after therapeutic targets [1, 2, 12, 13]. However, our understanding of the mechanisms driving their oncogenic function and effective strategies for targeting MYC oncogenic activity remains limited. Historically, MYC oncoproteins have been considered ‘undruggable’ due to their flexible protein structure [1, 2]. However, TFs like MYCN need to collaborate with cofactors to regulate gene transcription, and many of these cofactors possess druggable enzyme activity. Consequently, targeting MYCN cofactors represents one strategy to block MYCN’s tumor-promoting function. To date, druggable cofactors of MYCN that mediate its activity have not been comprehensively characterized.

Previous studies have reported that c-Myc interacts with various histone modification proteins to facilitate transcription [14], including Lysine Acetyltransferase 2 A (KAT2A, also known as GCN5) [15–19]. KAT2A serves as the catalytic subunit within the STAGA (SPT3-TAF(II)31-GCN5L acetyltransferase), SAGA (SPT-ADA-GCN5 acetyltransferase), or ATAC (Ada-Two-A-Containing) complex [17, 20–23] and acts as a histone acetyltransferase (HAT), which plays a pivotal role in the epigenetic landscape and chromatin modification. Additionally, KAT2A is capable of acetylating and stabilizing various non-histone substrates including c-Myc, Ezh2, E2A-PBX1, and HIF1a [24–26]. As an epigenetic regulator, KAT2A’s role in cancer has gained increased attention [20, 24, 27]. MYCN and KAT2A regulate overlapping transcription programs in neural stem cells [28]. Our recent MYCN interactome assay identified that KAT2A interacts with MYCN in NB cells [11]. Here we postulate a potential role for KAT2A in MYCN-mediated transcription and the potential for KAT2A inhibition as an anti-MYCN therapy for neuroblastoma.

In this study, we combine genome-wide approaches including RNA-seq and ChIP-seq to investigate the cooperation between MYCN and KAT2A on gene transcriptional regulation. We find that MYCN recruits KAT2A to activate canonical MYC targets including ribosome biogenesis genes and RNA processing genes. We identify that MYCN directly activates KAT2A transcription, while KAT2A acetylates MYCN and increases MYCN protein stability. Our study demonstrates that targeting KAT2A with a Proteolysis Targeting Chimera (PROTAC) degrader decreases MYCN activity and inhibits NB cell proliferation. This supports an approach of targeting MYCN cofactors as a MYCN targeting strategy.

## Results

### Genome-wide co-localization of MYCN and KAT2A

**Fig. 1:**
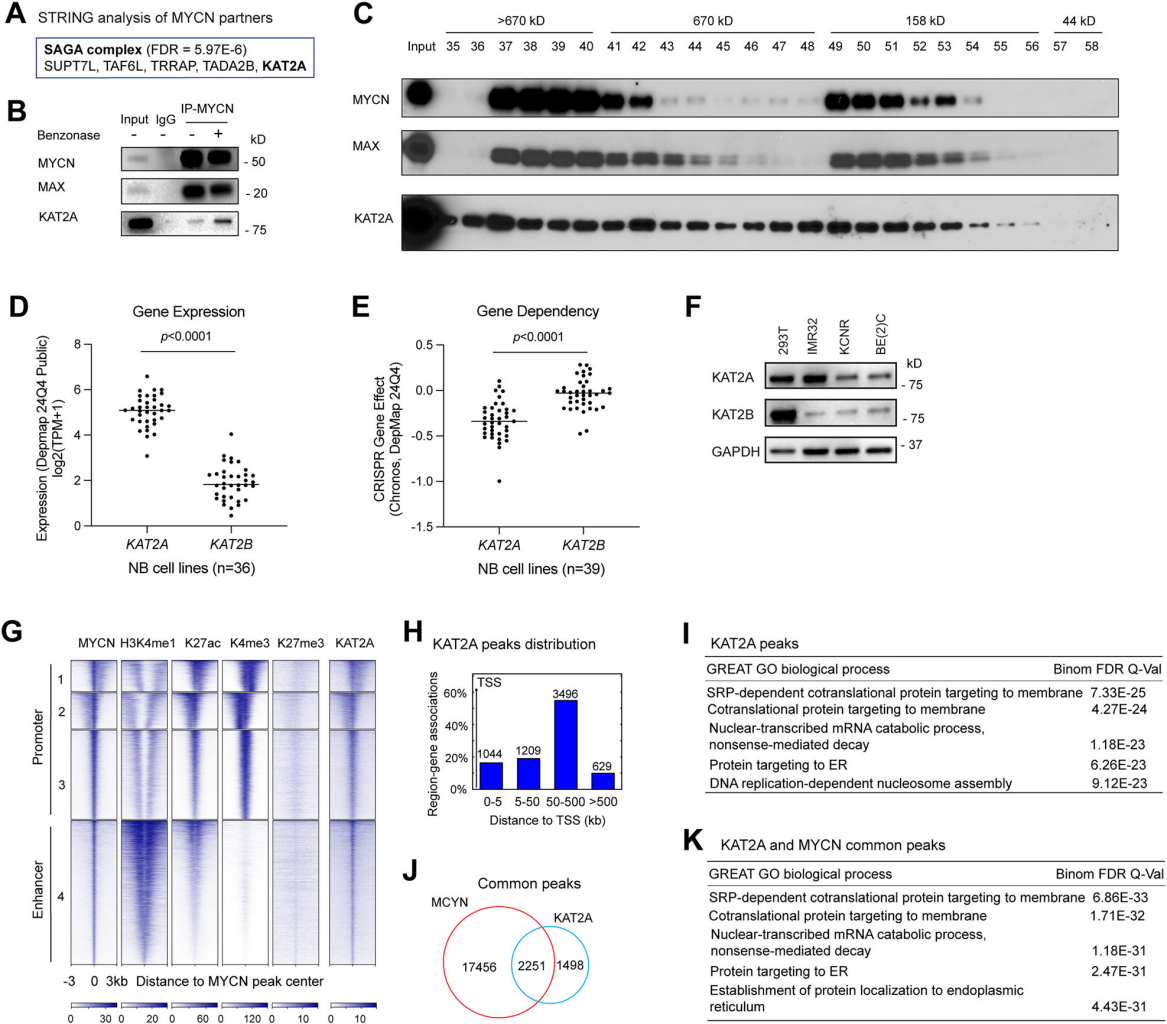
KAT2A is associated with MYCN. **A** STRING analysis of MYCN protein partners in the “chromatin organization” category shows a significant enrichment of the subunits of the SAGA complex. **B** The IMR32 whole cell extracts are used for co-IP with anti-MYCN antibody. Western blot assay is used to confirm the pulldown of MYCN cofactors KAT2A and MAX. **C** IMR32 cell extracts were fractionated through a gel filtration column, and selected fractions were then resolved by SDS-PAGE, and western blotting was performed to identify indicated proteins. **D** Comparison of *KAT2A* and *KAT2B* mRNA levels in the 36 NB cell lines (queried from depmap.org). **E** Determine *KAT2A* and *KAT2B* dependencies in the 39 NB cell lines by analyzing data from genome-wide cancer genetic vulnerability CRISPR screens on DepMap. **F** Western blot assays show the protein levels of KAT2A and KAT2B in HEK293T cell line and MYCN-amplified NB cell lines. **G** *K*-Means clustering of ChIP-seq results of MYCN, histone marks, and KAT2A around MYCN binding sites of NB cell line IMR32 (±3 kb). **H** GREAT peak distribution shows that approximately 20% of KAT2A peaks derived from IMR32 cells are within 5 kb from TSS (transcription start site), while 80% of KAT2A peaks are 5 kb away from TSS. **I** GREAT Gene Ontology (GO) analysis indicates that KAT2A binding sites associated genes are enriched in protein synthesis and RNA processing. **J** ChIPPeakAnno analysis shows the number of common and unique peaks between MYCN and KAT2A. **K** GREAT Gene Ontology (GO) analysis indicates that KAT2A and MYCN overlapped binding sites associated genes are enriched in protein synthesis and RNA processing. Data information: In panels (**D** and **E**), the sample sizes (number of NB cell lines) are displayed on the graph. The middle solid lines indicate the mean. Statistical differences were determined using a two-sided unpaired Student’s *t*-test.

Our previous MYCN interactome assay identified many MYCN protein partners in NB cells [11]. Gene ontology (GO) biological processes analysis of these proteins using the DAVID tool showed enrichment in various categories such as nuclear acid metabolic process, DNA repair, and chromatin organization (Supplementary Table 1). To identify protein complexes that could potentially cooperate with MYCN to modify histones and remodel chromatin to regulate gene transcription, we focused on those 80 proteins involved in chromatin organization (Supplementary Table 1) using the STRING protein-protein interaction tool. We found several subunits of the SAGA complex (Fig. 1A) or SAGA-type complex (Fig. S1A), and other protein complexes (Supplementary Table 1) interact with MYCN in *MYCN*-amplified NB cell line IMR32. The SAGA complex has histone acetyltransferase activity and is druggable, making it a promising target for anti-cancer therapies. As the catalytic subunit of the STAGA complex, SAGA complex, and ATAC complex [15, 23], we focused on KAT2A to delineate its role in MYCN regulation of gene transcription. Coimmunoprecipitation (Co-IP) studies indicated the interaction between MYCN and MAX or KAT2A occurs independent of nucleic acid since treatment of the samples with benzonase did not diminish the protein-protein interaction (Fig. 1B). Size exclusion assay of cell lysates from IMR32 cells showed that both MAX and KAT2A co-eluted with MYCN in the early fractions (≥670 kD), as well as later fractions (158 kD) (Fig. 1C). This suggested that the majority of MYCN protein is part of a large macromolecular complex containing both KAT2A and MAX.

To determine whether KAT2A is associated with ATAC complex in NB cells, we performed KAT2A co-IP and western blot assays. We found that both SAGA complex-specific subunit TADA2B and ATAC complex-specific subunit TADA2A interact with KAT2A (Fig. S1B), indicating that KAT2A is potentially associated with both SAGA and ATAC complexes in NB. These findings suggest that MYCN may cooperate with all these protein complexes in regulating gene transcription.

The KAT2A’s paralog, KAT2B, commonly known as P300/CBP-associated factor (PCAF), has a largely mutually exclusive pattern of expression with KAT2A [23]. Redundant and overlapping functions exist between the KAT2A and KAT2B have been discovered in mice and zebrafish [29–31]. Moreover, KAT2A and KAT2B are found in the SAGA complexes in a mutually exclusive manner [32]. To investigate the expression pattern of KAT2A and KAT2B in NB, we explored the DepMap online tool (https://depmap.org/portal/). We found that KAT2A mRNA levels are high while KAT2B mRNA levels are low (Fig. 1D). Moreover, the gene dependency assay indicates that KAT2A exhibits notable dependency in nearly 50% of NB cell lines (threshold of −0.4), while KAT2B exhibits notable dependency in only 2 out of 39 NB cell lines (Fig. 1E). Western blot results showed that KAT2A protein levels in *MYCN*-amplified NB cell lines are comparable to those in the “normal” cell line HEK 293 T, while the KAT2B protein levels are lower (Fig. 1F). These findings suggest that KAT2A plays a more prominent role in NB cell lines than KAT2B.

Although KAT2B was not identified as a MYCN protein partner in our interactome assay, we directly assessed whether MYCN interacts with KAT2B by performing a MYCN co-IP and assessing KAT2A/B using isoform-specific antibodies. Consistent with the mass spectrometry results, we detected the pulldown of KAT2A but not KAT2B using an anti-MYCN antibody (Fig. S1C). The absence of MYCN-KAT2B interaction could be due to the extremely low expression of KAT2B in NB cells.

To identify MYCN and KAT2A DNA binding sites, we performed genome-wide ChIP-seq analysis for MYCN and KAT2A in IMR32 cells and compared the ChIP-seq results of histone marks we generated in our previous study (GSE208424) [11]. *K*-Means clustering of histone marks with MYCN and KAT2A ChIP-seq around MYCN binding sites (±3 kb) showed that KAT2A co-localizes with MYCN at promoters enriched with H3K4me3 and H3K27ac signals, as well as with MYCN at enhancers, marked by H3K4me1 and H3K27ac signals (Fig. 1G). GREAT gene ontology (GO) analyses of each cluster (Supplementary Table 2) showed that genes associated with cluster 1 and cluster 3 are enriched in RNA processing; genes associated with cluster 2 are enriched in gene transcriptional regulation, while genes associated with cluster 4 are enriched in nervous system development and neuron differentiation (Fig. S1D). Consistent with the *K*-Means clustering analysis, GREAT peak distribution analysis showed that some 20% of KAT2A binding sites were within 5 kb of TSS while the majority (80%) of KAT2A binding sites were 5 kb away from TSS (Fig. 1H). GREAT GO analyses indicated that KAT2A-bound peaks associated genes were enriched in protein translation and RNA processing when focusing on the top-ranked biological processes (Fig. 1I). ChIPPeakAnno analysis showed that 75% of KAT2A binding sites overlapped with MYCN binding sites (Fig. 1J). These overlapping peak-associated genes were also enriched in protein translation and RNA processing (Fig. 1K). Notably, KAT2A peaks that did not overlap with MYCN were enriched in nucleosome and chromatin assembly (Fig. S1E). Collectively, these findings suggest that MYCN cooperates with KAT2A to regulate canonical MYC target genes involved in protein translation and RNA processing.

### MYCN directly activates *KAT2A* gene expression

**Fig. 2:**
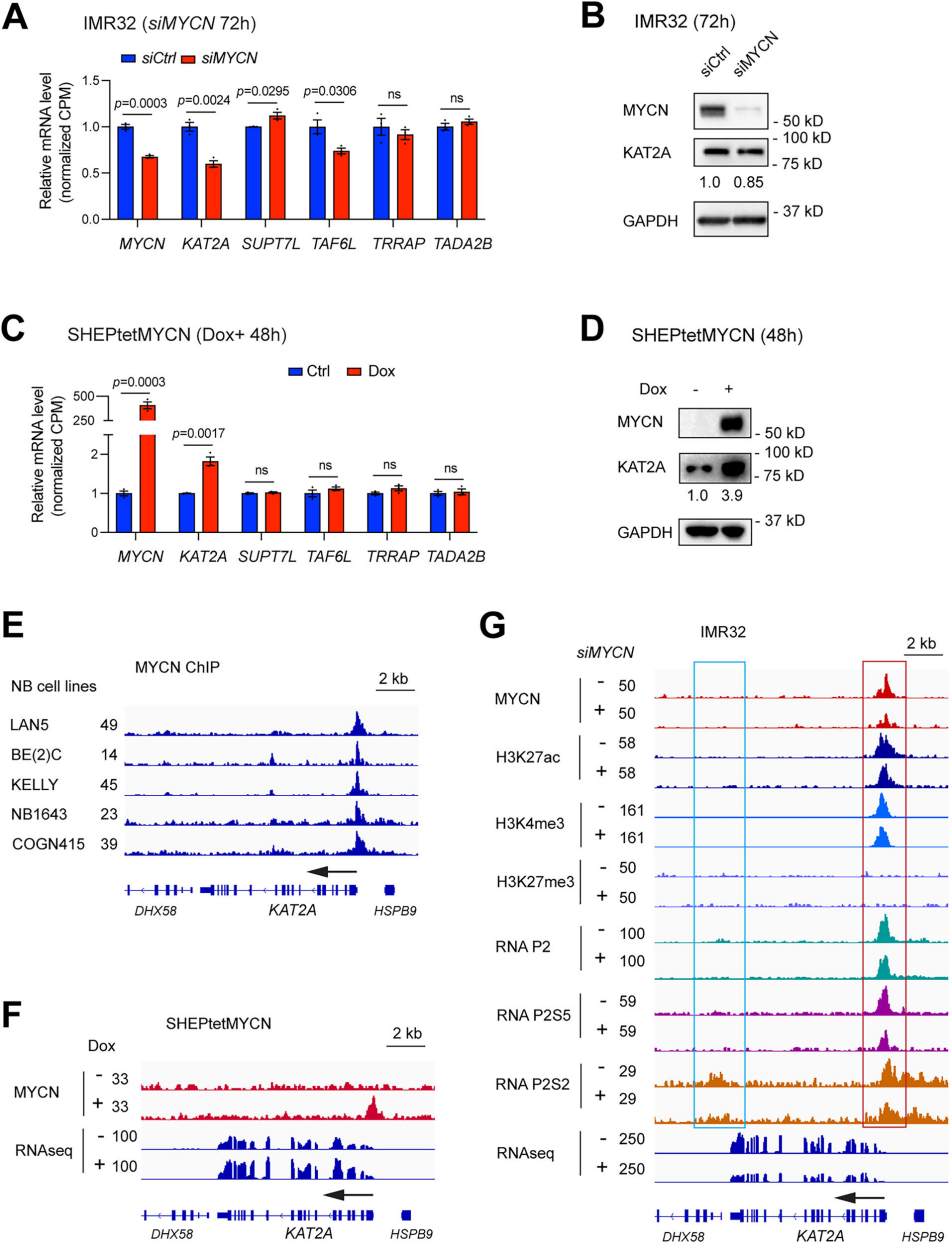
MYCN directly regulates KAT2A expression. **A** The effect of genetic silencing of *MYCN* in IMR32 cells on the mRNA expression levels of KAT2A and SAGA subunits was analyzed using RNA-seq data from three biological replicates. Data are presented as mean ± SEM. CPM: counts per million. ns: not significant. **B** Western blot assays show the effect of 72 h knockdown of *MYCN* in IMR32 cells on the expression of KAT2A at the protein levels. **C** The effect of *MYCN* overexpression in SHEP cells on the mRNA expression levels of KAT2A and SAGA subunits was analyzed using RNA-seq data from three biological replicates. Data are presented as mean ± SEM. CPM: counts per million. ns not significant. **D** Western blot assays show the effect of 48 h overexpression of *MYCN* in SHEP cells on the expression of KAT2A at the protein levels. **E** Signal tracks of the publicly available MYCN ChIP-seq data (GSE138295) show that MYCN binds to the promoter of the *KAT2A* gene in *MYCN*-amplified NB cell lines. **F** Signal tracks show gained MYCN peaks within the promoter of *KAT2A* when *MYCN* is overexpressed in SHEP cells. **G** The depletion of *MYCN* does not significantly affect H3K27ac and H3K4me3 signal intensity at the *KAT2A* gene locus but leads to a decrease of RNA pol II Ser5P signal at the transcription start site (TSS) (red box) and a decrease of RNA Pol II Ser2P signal within the region downstream of the poly-adenylation signal of KAT2A gene (cyan box).

To investigate the relationship between MYCN and KAT2A, we first investigated whether the loss or gain of *MYCN* affects *KAT2A* expression. We found that the silencing of *MYCN* led to a 40% decrease in *KAT2A* at the mRNA levels (Fig. 2A) detected by interrogating our previously published RNA-seq data (GSE183641) [33] and this is accompanied by a 15% decrease in KAT2A at the protein levels (Fig. 2B). Overexpression of *MYCN* in SHEP, an NB cell line with no endogenous *MYCN* expression, resulted in a significant upregulation in *KAT2A* mRNA levels (Fig. 2C) detected by interrogating our previously published RNA-seq data (GSE208424) [11] and protein levels (Fig. 2D). Unlike KAT2A, the mRNA levels of most of the rest SAGA subunits that interact with MYCN did not change when *MYCN* was silenced or overexpressed (Fig. 2A, C). Moreover, we found that the knockdown of *MYCN* in IMR32 cells significantly increased *KAT2B* mRNA levels, while overexpression of *MYCN* in SHEP cells led to a modest but not significant decrease in *KAT2B* expression (Fig. S2A, B). These results indicated that MYCN directly activates *KAT2A* but not *KAT2B* transcription.

By interrogating publicly available MYCN ChIP-seq data (GSE138295), we found that endogenous MYCN binds to the promoter of the *KAT2A* gene in all these *MYCN*-amplified NB cell lines (Fig. 2E). Consistently, ChIP-seq and RNA-seq data analysis indicated that MYCN binds to the promoter of *KAT2A* and upregulates *KAT2A* when it was overexpressed in SHEP cells (Fig. 2F). We next investigated the epigenome changes at the *KAT2A* gene locus to determine how the silencing of *MYCN* in IMR32 led to a decrease in *KAT2A* expression. By interrogating our previous MYCN ChIP-seq data (GSE208424) after MYCN perturbation [11], and performing additional RNA P2, P2S5, and P2S2 ChIP-seq experiments, we found that the depletion of *MYCN* did not significantly affect H3K27ac and H3K4me3 signal intensity but led to a decrease of RNA Pol II Ser5P signal at the transcription start site (TSS) (Fig. 2G, red box), and a decreased signal of RNA Pol II Ser2P was observed in the region downstream of the poly-adenylation signal of KAT2A (Fig. 2G, cyan box). This indicates that *MYCN* depletion reduces RNA Pol II initiation and elongation at the *KAT2A* gene locus.

### MYCN recruits KAT2A to bind both promoters and distal regulatory regions

TFs typically recruit cofactors to their binding site to regulate gene transcription. To investigate whether MYCN recruits KAT2A to its binding sites, we performed ChIP-seq experiments using anti-MYCN and KAT2A antibodies in control small interfering RNA (*siCtrl*) and *MYCN* siRNA (*siMYCN*) transfected IMR32 cells. ChIP-seq results showed that the knockdown of *MYCN* in IMR32 cells dramatically reduced KAT2A ChIP-seq peak number from 3749 to 830 (Supplementary Table 3). The silencing of *MYCN* resulted in a subtle (15%) decrease of KAT2A at the protein levels (Fig. 2B), which could partially contribute to the reduction of the KAT2A peak numbers in *siMYCN* cells.

**Fig. 3:**
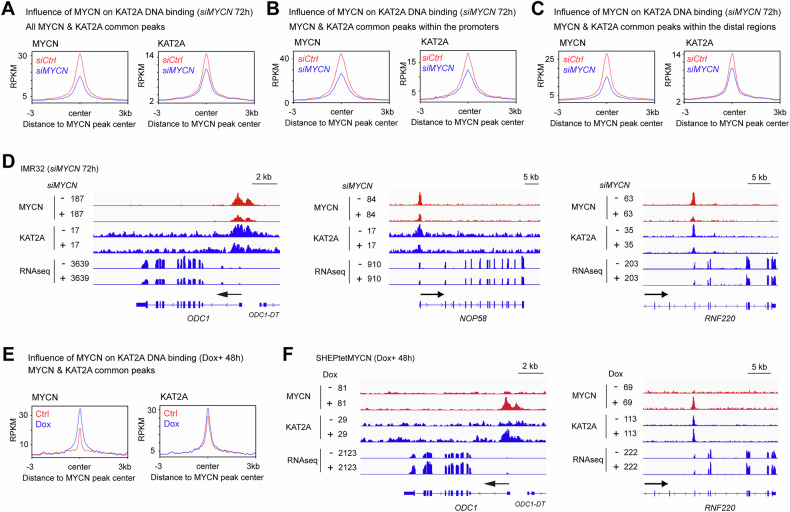
Silencing of *MYCN* alters genomic DNA binding of KAT2A. Metagene plots show that the knockdown of *MYCN* in IMR32 cells results in a decrease of average MYCN and KAT2A signal intensity at the MYCN peak center of (**A**), all the MYCN & KAT2A overlapped binding sites, or (**B**), the MYCN & KAT2A overlapped binding sites within the promoters, or (**C**), the MYCN & KAT2A overlapped binding sites within the distal regulatory regions. **D** Signal tracks show that the knockdown of *MYCN* results in a decrease in MYCN and KAT2A signals at the promoter of the *ODC1* gene and *NOP58* gene, as well as the distal regulatory region of the *RNF220* gene. **E** Metagene plots show that the overexpression of *MYCN* in SHEP cells increases average MYCN and KAT2A signal intensity at the MYCN peak center. **F** Signal tracks show that the overexpression of *MYCN* results in an increase in MYCN and KAT2A signals at the promoter of the *ODC1* gene, as well as the distal regulatory region of the *RNF220* gene.

By focusing on MYCN and KAT2A overlapped peaks, metagene plots showed that the knockdown of *MYCN* resulted in a decrease in average MYCN and KAT2A ChIP-seq signals at the MYCN peak center (Fig. 3A). C-Myc recruitment of KAT2A to promoter regions has been shown for a few genes by ChIP PCR analyses or the use of reporter assays [15, 17]. A recent study showed that c-Myc inhibitor treatment led to a loss of KAT2A binding at c-Myc-bound enhancers with no change in binding at promoters [19]. We annotated KAT2A and MYCN overlapped peaks using HOMER software to identify promoters and distal regulatory regions that are mostly considered enhancers. The knockdown of *MYCN* results in a decrease in the average MYCN and KAT2A ChIP-seq signals of the overlapping peaks at promoters (Fig. 3B). GREAT GO analysis indicated these peaks with reduced signals were associated with genes involved in protein translation and RNA processing (Fig. S3A). The knockdown of *MYCN* resulted in a decrease in the average KAT2A signal within distal regulatory regions (Fig. 3C), and these peaks were associated with genes involved in nervous system development (Fig. S3B). For example, signal tracks are shown for the promoters of the canonical MYCN target gene *ODC1* and the ribonucleoprotein *NOP58* coding gene as well as within the distal regulatory region of the *RNF220* gene (Fig. 3D). Conversely, when *MYCN* is overexpressed in SHEP cells, metagene plots showed an increase in average KAT2A ChIP-seq signals of the MYCN/KAT2A overlapping peaks (Fig. 3E). For example, signal tracks showed an increase of KAT2A signals within the promoter of *ODC1* and *NOP58* gene (Fig. 3F, left panel; Fig. S3C), as well as an increase in KAT2A signal within the distal regulatory region of *RNF220* gene (Fig. 3F, right panel). Interestingly, although *MYCN* upregulated KAT2A protein levels in SHEP cells (Fig. 2D), metagene plots showed that the overexpression of *MYCN* led to only slight increases in KAT2A ChIP-seq signals when focusing on all KAT2A peaks (Fig. S3D). This increase was not as pronounced as the rise in KAT2A ChIP-seq signals at the MYCN/KAT2A overlapping peaks (Fig. 3E). SHEP cells express c-MYC, and we postulate that the high levels of c-MYC in SHEP cells already assist KAT2A in binding to the c-MYC binding sites, leading to high basal KAT2A ChIP-seq signals. Therefore, the increased KAT2A binding is dominant at newly acquired MYCN binding sites when *MYCN* is overexpressed.

A recent study focused on SAGA in NB showed that TADA2B (specific to the SAGA complex) colocalizes with MYCN at the promoters [34], supporting the binding of SAGA at proximal sites. However, we cannot rule out the binding of the ATAC complex since KAT2A also interacts with ATAC-specific subunit TADA2A (Fig. S1B). Nevertheless, our findings demonstrate that, at a genome-wide level, MYCN recruits KAT2A to both promoters and enhancers to regulate gene transcription.

### KAT2A increases MYCN protein stability

**Fig. 4:**
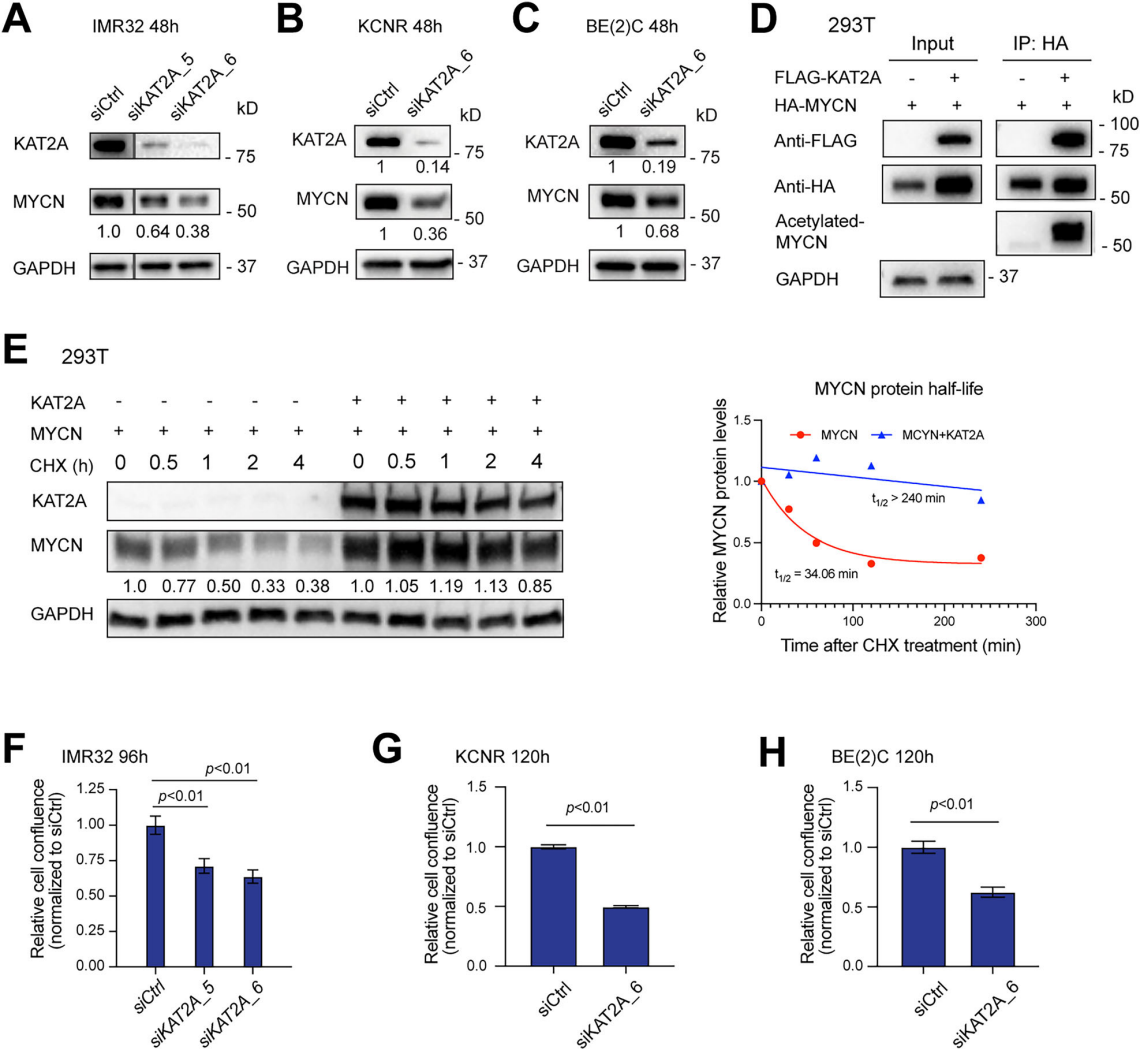
KAT2A regulates MYCN stability. **A–C** Knockdown of *KAT2A* in *MYCN* amplified NB cell lines for 48 h results in a decrease in KAT2A and MYCN protein levels detected by western blotting assay. **D** 293 T cells were either co-transfected with HA-MYCN and FLAG-KAT2A or with FLAG-KAT2A alone for 24 h. Whole-cell lysates were co-immunoprecipitated with HA-Tag antibody and analyzed by Western blotting; the blot was probed with anti-HA, anti-FLAG, and anti-acetylated lysine antibodies. Blot shows an increase in N-Myc acetylation in the co-transfected condition. **E** 293 T cells were either co-transfected with HA-MYCN and FLAG-KAT2A or HA-MYCN alone for 24 h. Cells were then treated with 50 μg/mL cycloheximide (CHX) and harvested at set intervals. Whole-cell lysates were analyzed by Western blotting; the blot was probed for KAT2A and MYCN. MYCN protein levels are more stable over time in the co-transfected condition (left panel). Protein levels from the western blot were quantified using ImageJ software, normalized to GAPDH levels, and plotted as an exponential decay function using PRISM software (right panel). **F-H** Genetic silencing of *KAT2A* in MYCN amplified NB cell lines leads to a decrease in cell proliferation detected by the IncuCyte confluence assay.

KAT2A acetylates and stabilizes various non-histone substrates including c-Myc, Ezh2, and HIF1a [24, 25]. To investigate whether KAT2A regulates MYCN protein stability, we silenced *KAT2A* with *siRNAs* in NB cells. Knockdown of *KAT2A* resulted in an 80–90% decrease of KAT2A and this was associated with a 30–60% decrease of MYCN protein levels in 3 different MYCN amplified NB cell lines (Fig. 4A–C). Notably, we also observed that the silencing of *KAT2A* resulted in a 20% decrease in MYCN mRNA levels based on RNA-seq results (Supplementary Table 4), which could partially contribute to the reduction of MYCN protein. Additionally, consistent with its role as a histone acetyltransferase, we found that the knockdown of *KAT2A* led to a decrease in the protein levels of its target H3K9ac (Fig. S4A). To investigate whether KAT2A regulates c-MYC protein stability in NB, we knocked down *KAT2A* in a non-*MYCN* amplified NB cell line SK-N-AS, which expresses c-MYC instead of MYCN. As expected, western blot results indicated that the silencing of *KAT2A* in SK-N-AS cells resulted in a decrease in c-MYC protein levels (Fig. S4B).

To investigate whether KAT2A acetylates MYCN protein, we co-transfected HEK293T cells with HA-tagged MYCN (HA-MYCN) alone or in combination with FLAG-tagged KAT2A (FLAG-KAT2A) and performed co-immunoprecipitation using an anti-HA antibody. Cells transfected with both KAT2A and MYCN had increased MYCN protein levels compared to those transfected with MYCN alone (Fig. 4D, left) and in these cells, acetylation of MYCN was detected by using an anti-acetyl antibody in cells transfected with both KAT2A and MYCN but not in cells transfected with MYCN alone (Fig. 4D, right).

To confirm the mediation of MYCN protein stability by KAT2A, cycloheximide (CHX) treatment was administered to HEK293T cells either co-transfected with MYCN and KAT2A or MYCN alone. We found that in the presence of KAT2A, the stability or half-life of MYCN increased from 34 min to 240 min (7-fold) (Fig. 4E). The finding that KAT2A stabilizes MYCN protein suggests that KAT2A may mediate MYCN oncogenic activity such as regulating cell proliferation. Consistent with this hypothesis, silencing of *KAT2A* resulted in a 30–50% decrease in cell proliferation compared to cells transfected with *siCtrl* in IMR32, KCNR, and BE(2)C cell lines (Fig. 4F–H).

To investigate whether KAT2B plays a role in regulating MYCN protein stability in NB, we knocked down *KAT2B* in IMR32 cells and performed a western blot assay. We found that the depletion of KAT2B did not decrease the protein levels of MYCN and H3K9ac, nor did it affect KAT2A protein levels (Fig. S4C). Interestingly, when we knocked down *KAT2A* in IMR32 cells, we observed an increase in KAT2B protein levels (Fig. S4D) and a 1.4-fold increase in KAT2B mRNA levels (detected by RNA-seq, Supplementary Table 4), supporting the compensatory expression of KAT2B when *KAT2A* is depleted.

By exploring the predictability function in DepMap, we observed that low levels of *KAT2B* are significantly positively correlated with the effects of *KAT2A* CRISPR knockout across all NB cell lines (Fig. S4E), while low levels of *KAT2A* are not correlated with the effects of *KAT2B* CRISPR knockout (Fig. S4F). The observation that NB cell lines with lower *KAT2B* expression appear to be more sensitive to the loss of *KAT2A* further suggests a redundant and compensatory role of KAT2B in NB tumorigenesis.

### The depletion of KAT2A antagonizes MYCN-mediated gene transcription regulation

**Fig. 5:**
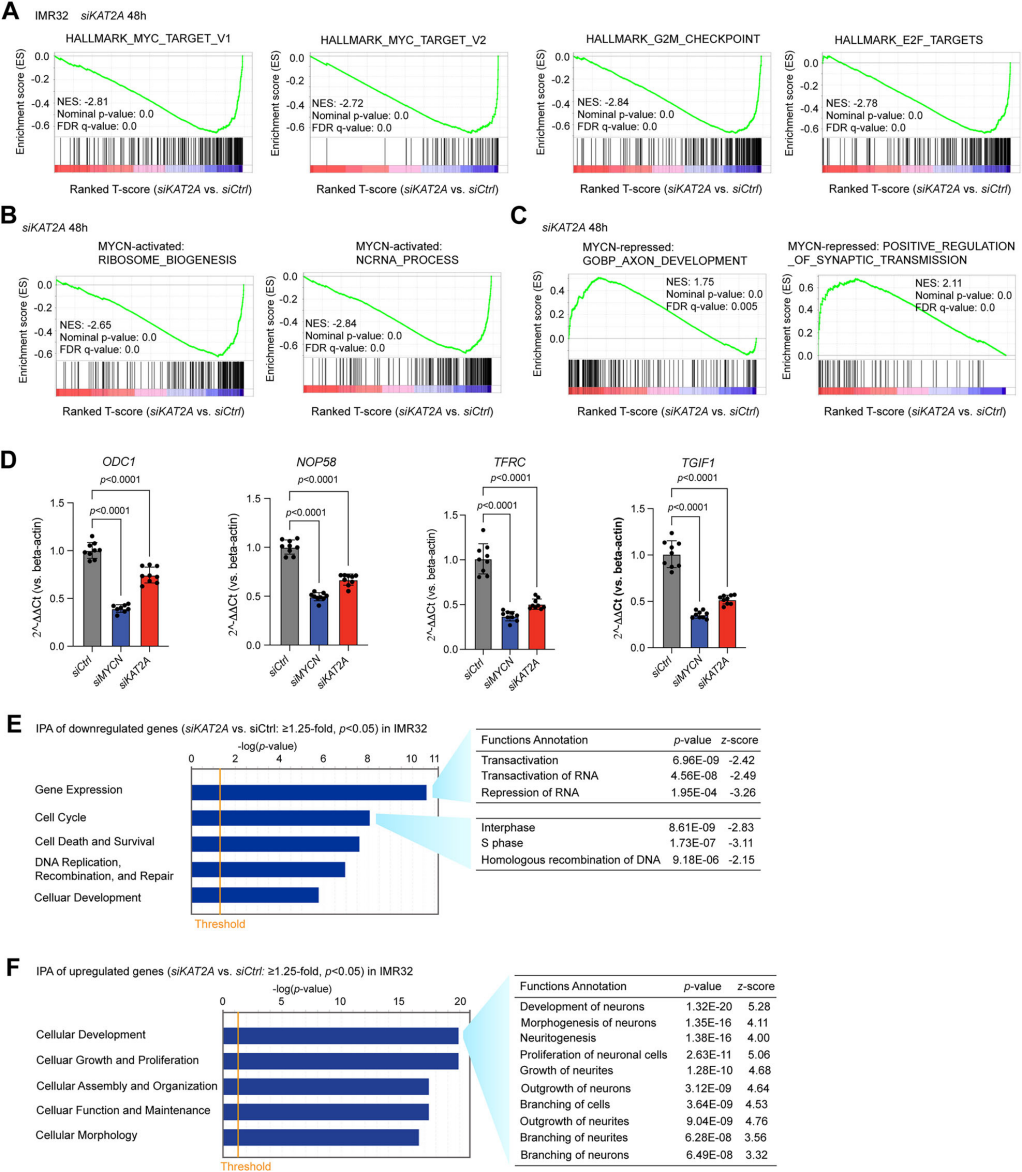
The depletion of *KAT2A* antagonizes MYCN-mediated gene transcription regulation. **A** GSEA shows that the silencing of *KAT2A* in IMR32 cells for 48 h results in a significant negative enrichment of MYC targets, G2M checkpoint genes, and E2F targets. **B** GSEA shows that the silencing of *KAT2A* results in a significant negative enrichment of genes involved in RNA processing and ribosome biogenesis that are known to be activated by MYCN. **C** GSEA shows that the silencing of *KAT2A* results in a significant positive enrichment of genes neuronal genes that are known to be repressed by MYCN. **D** Real-time PCR results show that the knockdown of *MYCN* or *KAT2A* using *siRNAs* for 48 h significantly reduces the mRNA levels of *ODC1, NOP58, TFRC*, and *TGIF1* compared to the *siRNA* control (*siCtrl*) (*n* = 9). Data are presented as mean ± SD. **E** Ingenuity Pathway Analysis (IPA) indicates that genes down-regulated after genetic silencing of *KAT2A* are involved in regulating gene expression and cell cycle progression. **F** IPA indicates that genes up-regulated after genetic silencing of *KAT2A* are involved in regulating neuronal differentiation.

To investigate whether KAT2A functions as a cofactor of MYCN to regulate the expression of MYCN target genes, we evaluated the transcriptional profiles after knocking down *KAT2A* in IMR32 cells for 48 h. Gene set enrichment analysis (GSEA) of the RNA-seq data (Supplementary Table 4) indicated that the silencing of *KAT2A* resulted in a significantly negative enrichment of MYC targets, as well as G2M checkpoint regulators and E2F targets (Fig. 5A). Previously, we have defined MYCN-activated ribosome biogenesis, RNA processing, ribosome formation, and cytoplasmic translation gene signatures, as well as MYCN-repressed neuronal gene signatures that are involved in axon development and positive regulation of synaptic transmission [11]. GSEA results showed that the silencing of *KAT2A* resulted in a significant negative enrichment of “MYCN activated canonical MYC targets” gene sets such as genes involved in ribosome biogenesis and RNA processing (Fig. 5B), as well as a significant positive enrichment of “MYCN repressed neuronal genes” gene sets such as those involved in axon development and positive regulation of synaptic transmission (Fig. 5C). Furthermore, real-time PCR validation showed that MYCN and KAT2A targets *ODC1, NOP58, TFRC*, and *TGIF1* identified by RNA-seq were down-regulated after the knockdown of either *MYCN* or *KAT2A* (Fig. 5D). Ingenuity Pathway Analysis (IPA) showed that *KAT2A* depletion led to negative enrichment of genes involved in regulating gene expression and cell cycle progression (Fig. 5E) and positive enrichment of genes involved in neuronal differentiation (Fig. 5F).

### Targeting KAT2A with PROTAC degrader suppresses NB cell proliferation

**Fig. 6:**
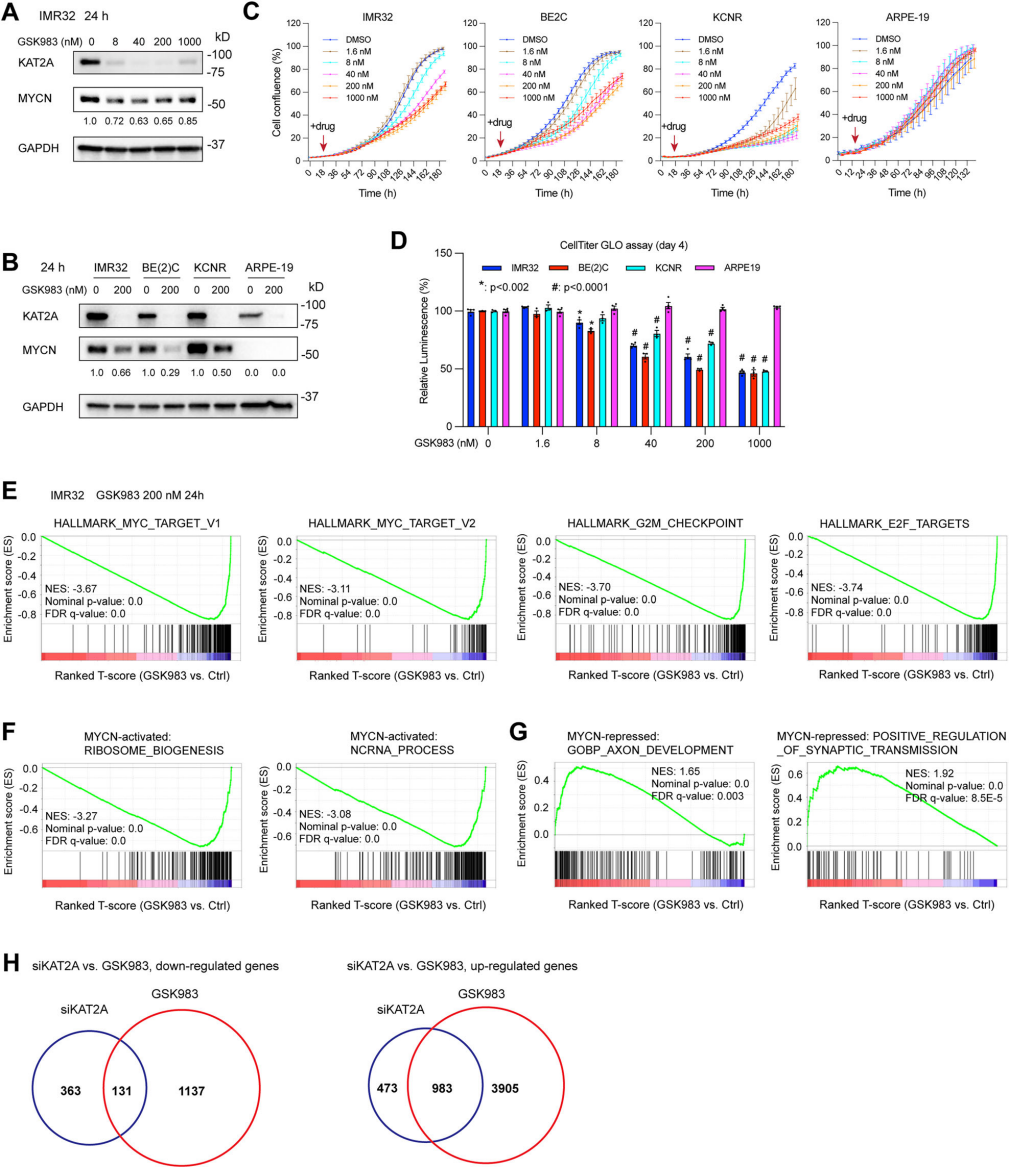
Targeting KAT2A with PROTAC degrader suppresses NB cell proliferation. **A, B** KAT2A PROTAC degrader GSK983 treatment of NB cells leads to the reduction of KAT2A, which is accompanied by a decrease of MYCN at protein levels. **C** Targeting KAT2A with GSK983 leads to a decrease in cell proliferation in NB cell lines but not normal cell line ARPE-19 based on IncuCyte cell confluence assay (*n* = 4 for IMR32 and ARPE19; *n* = 3 for BE(2)C and KCNR), and **D**, based on CellTiter-Glo cell viability assay (*n* = 4 for IMR32 and ARPE19; *n* = 3 for BE(2)C and KCNR). Data are presented as mean ± SEM. **E** GSEA shows that the targeting of *KAT2A* with GSK983 in IMR32 cells for 24 h results in a significant negative enrichment of MYC targets, G2M checkpoint genes, and E2F targets. **F** GSEA shows that the targeting of *KAT2A* with GSK983 results in a significant negative enrichment of genes involved in RNA processing and ribosome biogenesis that are activated by MYCN. **G** GSEA shows that the targeting of *KAT2A* with GSK983 results in a significant positive enrichment of genes neuronal genes that are repressed by MYCN. **H** Venn diagram analysis of the genes down-regulated and up-regulated by genetic silencing of *KAT2A* and PROTAC-mediated KAT2A degradation. Approximately 26% of genes down-regulated after genetic silencing of KAT2A are also found to be down-regulated after treatment with the KAT2A PROTAC degrader GSK983 (left panel), while approximately 67% of genes up-regulated after genetic silencing of KAT2A are also up-regulated after GSK983 treatment (right panel). Genes are considered differentially expressed when there is a ≥ 1.25-fold increase or decrease in mRNA levels with *p* < 0.05. Data information: In panel (**D**), data are presented as mean ± SE. The results are a representative set of two independent experiments. Statistical differences were assessed using one-way ANOVA. In panels (**E**–**G**), GSEA uses permutation testing to estimate the significance of the enrichment score.

To recapitulate our genetic inhibition findings, we pharmaceutically targeted KAT2A using the inhibitor GSK983, which specifically targets KAT2A/KAT2B for proteolytic degradation [35]. Treatment of IMR32 cells with the KAT2A degrader molecule significantly decreased KAT2A protein levels as expected (Fig. 6A). Within 24 h of KAT2A depletion there was a 35–80% reduction of MYCN protein levels in several NB *MYCN* amplified cell lines (Fig. 6A, B). GSK983 treatment of NB cell lines resulted in a decrease in cell proliferation as demonstrated by IncuCyte cell confluence assay, CellTiter-Glo cell viability assay, and the Incucyte cell imaging analysis (Figs. 6C, D and S5A–C). In a non-tumorigenic human epithelial cell line ARPE-19, GSK983 treatment depleted KAT2A (Fig. 6B) but did not affect cell proliferation (Figs. 6C, D and S5D).

RNA-seq data analysis of IMR32 cells treated with GSK983 for 24 h (Supplementary Table 5) indicated that the depletion of KAT2A led to a negative enrichment MYC targets, as well as G2M checkpoint regulators and E2F targets (Fig. 6E). Moreover, the depletion of KAT2A resulted in a significant negative enrichment of “MYCN activated canonical MYC targets” gene sets such as genes involved in ribosome biogenesis and RNA processing (Fig. 6F), as well as a significant positive enrichment of “MYCN repressed neuronal genes” gene sets such as genes involved in axon development and positive regulation of synaptic transmission (Fig. 6G).

Next, we compared the differentially expressed genes identified by knocking down *KAT2A* and PROTAC-mediated KAT2A degradation. RNA-seq results indicated that 494 genes were down-regulated, and 1268 genes were up-regulated when *KAT2A* was knocked down. More dramatically, 1456 genes were down-regulated, and 4888 genes were up-regulated when KAT2A was depleted through the PROTAC degrader GSK983 treatment (Supplementary Table 4 and Table 5). Furthermore, we performed a Venn diagram analysis of these gene lists. We found that approximately 26% of genes down-regulated after genetic silencing of *KAT2A* were also down-regulated after treatment with GSK983 (Fig. 6H, left panel; Supplementary Table 6), while approximately 67% of genes up-regulated after genetic silencing of *KAT2A* were also up-regulated after GSK983 treatment (Fig. 6H, right panel; Supplementary Table 6). PROTAC-mediated degradation of KAT2A has a much more dramatic effect on gene transcription than genetic silencing of *KAT2A*, possibly because the PROTAC degrader can remove both KAT2A and its paralog KAT2B [34, 35], whereas the genetic silencing of *KAT2A* increased KAT2B (Fig. S4D). These findings support the role of KAT2A in mediating MYCN’s transcriptional activity and targeting both KAT2A and KAT2B with the dual PROTAC degrader could be a promising approach for treating NB.

## Discussion

**Fig. 7:**
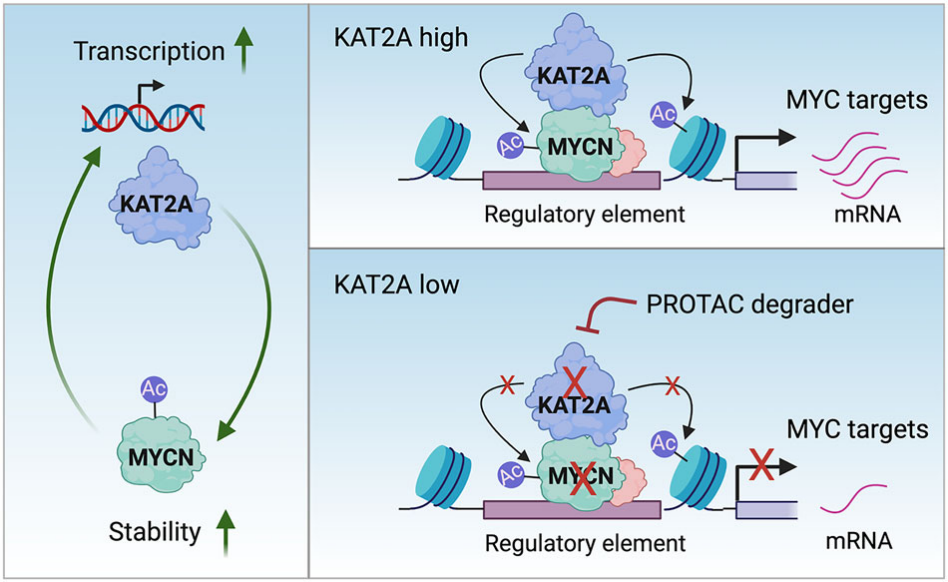
Schematic diagram illustrating the cooperation between MYCN and KAT2A. MYCN directly activates KAT2A transcription, while KAT2A acetylates and stabilizes MYCN protein. MYCN recruits KAT2A to activate its targets and targeting KAT2A through PROTAC degrader antagonizes MYCN-mediated gene transcription regulation (Created with BioRender.com).

In this study, we uncovered a novel mechanism by which the oncoprotein MYCN drives gene expression in NB via a feedforward loop with the coactivator KAT2A. Specifically, MYCN recruits KAT2A to occupy chromatin at the genome-wide level, facilitating the expression of many pro-growth oncogenes including KAT2A. Besides its canonical histone acetyltransferase activity, KAT2A also acetylates and stabilizes the MYCN protein, suggesting a multi-pronged mechanism by which this acetyltransferase enhances MYCN transcriptional activation. Importantly, targeting KAT2A through a drug-like small molecule degrader antagonizes MYCN’s activity (Fig. 7). Our findings shed light on the intricate regulatory network underlying MYCN-mediated tumorigenesis and provide potential therapeutic strategies for countering MYCN’s oncogenic activity.

Our study provides compelling evidence for the existence of a feedforward loop between MYCN and KAT2A. Specifically, MYCN directly activates *KAT2A* transcription by binding to its promoter and enhancing RNA Pol II initiation and elongation. In turn, KAT2A acetylates and stabilizes the MYCN protein. This regulatory circuitry ensures a sustained and potent activation of MYCN, contributing to its role as a key driver of malignancy in NB. Our ChIP-seq analysis reveals the genome-wide co-localization of MYCN and KAT2A, providing a comprehensive view of their regulatory roles. The functional significance of this co-localization is highlighted by the enrichment of genes involved in protein translation and RNA processing, processes that are critical for the growth and survival of cancer cells. MYCN directly activates canonical MYC targets, including genes involved in protein translation and RNA processing, while simultaneously repressing neuronal differentiation genes [11]. Our RNA-seq analysis following *KAT2A* knockdown reveals a substantial impact on MYCN target genes, particularly down-regulating those involved in ribosome biogenesis and RNA processing. Conversely, a subset of genes associated with neuronal differentiation are upregulated, highlighting the role of the MYCN-KAT2A axis in driving a malignant neuroblastoma phenotype. Notably, ChIP-seq results indicate that KAT2A predominantly binds to genomic loci associated with protein translation and RNA processing, with limited binding to neuronal differentiation genes. This suggests that KAT2A functions as a coactivator of MYCN, directly facilitating MYCN’s role in activating genes involved in protein translation and RNA processing while indirectly repressing neuronal differentiation genes through the stabilization of MYCN protein.

One limitation of this study is that we did not determine which lysine is acetylated on MYCN and the underlying mechanism by which acetylation increases MYCN protein stability. Moreover, we have not determined whether MYCN directly interacts with KAT2A or through TRRAP. c-MYC directly binds both KAT2A and TRRAP [17]. Based on the high homology between MYCN and c-MYC, we believe MYCN also binds both KAT2A and TRRAP directly. It has been reported that KAT2A acetylates mouse c-Myc at lysines 323 and 417 to increase its protein stability [25], but the mechanism remains unclear. Similar lysine positions are present in human MYCN, suggesting that these two lysines in MYCN could potentially be acetylated by KAT2A. We postulate the following potential mechanisms that may contribute to the increase in MYCN protein stability: 1. Acetylation blocks ubiquitination sites on lysine residues, preventing the MYCN from being marked for degradation by the ubiquitin-proteasome system; 2. Acetylation induces conformational changes in MYCN, potentially enhancing its stability by promoting a more stable structure; or 3. Acetylation affects the binding affinity of MYCN for its interaction partners, protecting MYCN from degradation by stabilizing beneficial interactions.

We used GSK983, a small molecule degrader optimized for drug-like properties, to target the transcriptional coactivator KAT2A in MYCN-driven neuroblastoma. This molecule stands in contrast to previous putative small molecule inhibitors of KAT2A/KAT2B, such as butyrolactone and garcinol, which have been found to function as promiscuous electrophiles and aggregators, respectively. As a bifunctional molecule, GSK983 has the advantage of targeting both catalytic and non-catalytic properties of KAT2A. However, it is also important to note that the development of such inhibitors can present challenges from a pharmacokinetic, pharmacodynamic, and formulation perspective. It remains to be determined whether the degradation of KAT2A affects the integrity of the SAGA complex or any non-catalytic roles the latter may play in MYCN-mediated transcriptional activation. Another question is whether inhibitors of the KAT2A bromodomain or catalytic domain, should they be developed, would have similar effects as the degrader molecule.

MYCN has historically presented a challenge for targeted therapy due to its flexible protein structure. Our study highlights the potential of KAT2A as a viable therapeutic target for NB. Genetic silencing of *KAT2A* and pharmacological degradation of KAT2A using a PROTAC degrader GSK983 result in a decrease in MYCN activity and inhibit NB cell proliferation. This is consistent with a recent report that targeting KAT2A/KAT2B with a PROTAC degrader, GSK-699, suppresses NB tumor growth [34]. Identifying KAT2A as a critical mediator of MYCN’s oncogenic activity opens a promising avenue for developing anti-MYCN therapies. Notably, depleting KAT2A through a genetic or PROTAC approach does not completely halt cell growth, mirroring the effect of genetic silencing of *MYCN* [11]. This suggests that KAT2A inhibition could be part of combination therapy to effectively target MYCN-driven malignancy, possibly in conjunction with small molecules that could induce synthetic lethality in MYCN-addicted NB.

In conclusion, our study has unveiled a previously unrecognized feedforward loop between MYCN and KAT2A that orchestrates an oncogenic transcriptional program in neuroblastoma. These findings not only advance our knowledge of MYCN biology but also present a promising therapeutic strategy for the treatment of high-risk neuroblastoma, a disease with limited treatment options and poor prognosis. Further investigation and clinical development of KAT2A-targeted therapies hold the potential to make a significant impact on the management of this aggressive childhood cancer.

## Materials and methods

### Cell culture

Human neuroblastoma (NB) cell lines IMR32, SK-N-BE(2)C (BE(2)C), SMS-KCNR (KCNR), and SK-N-AS (AS) were obtained from the cell line bank of the Pediatric Oncology Branch of the National Cancer Institute and were genetically verified. ARPE-19 cell line, a spontaneously arising human retinal pigment epithelial (RPE) line, and the HEK293T cell line were both obtained from ATCC. All NB and ARPE-19 cell lines were cultured in RPMI-1640 complete medium, whereas HEK293T cells were cultured in DMEM complete medium supplemented with 10% fetal calf serum (FBS), 100 µg/mL streptomycin, 100 U/mL penicillin, and 2 mM L-glutamine. Cells were grown at 37 °C with 5% CO2. SHEPtetMYCN cells were established by infecting SHEP cells with lentiviral particles generated using the pLVX-TetOne-Puro-MYCN vector. The cells were selected with 0.65 µg/ml puromycin and maintained in RPMI-1640 complete medium. MYCN expression in SHEPtetMYCN was inducible with 0.25 µg/ml Dox treatment. All cell lines were confirmed to be free of mycoplasma contamination.

### PROTAC degrader GSK983

GSK983 was prepared by the published synthetic route [35]. Purity and identity of the product was determined by LC-MS prior to use in cell culture assays.

### Transient transfection

siRNA control (AllStars Negative Control siRNA, Catalog No. 1027281) and siRNAs targeting different genes (Hs_MYCN_2, Catalog No. SI00076293; Hs_KAT2A_5, Catalog No. SI03216507; Hs_KAT2A_6, Catalog No. SI04235756) were purchased from Qiagen or Santa Cruz Biotechnology. siKAT2B ON-TARGETplus SMARTpool was purchased from Dharmacon. siRNAs were transiently transfected into NB cells using Nucleofector electroporation (Lonza): solution L and program C-005 for IMR32; solution V and program A-030 for SK-N-AS, BE(2)C and KCNR. To transfect HEK293T cells, Lipofectamine 2000 cationic lipid reagent (Invitrogen) was used according to the manufacturer’s protocol.

### Cell proliferation and viability assay

To evaluate cell proliferation, 4 × 10^3^ IMR32, 2.5 × 10^3^ BE(2)C, 4 × 10^3^ KCNR, and 1.5 × 10^3^ ARPE-19 cells were seeded into standard flat-bottom, cell culture grade 96-well plates and incubated overnight. The following day, the cells were treated with varying doses of the KAT2A PROTAC degrader GSK983 or a DMSO control, with three to four technical replicates for each cell line. Growth kinetics in one plate was monitored through the IncuCyte ZOOM system (Essen BioScience), with the integrated confluence algorithm serving as a surrogate measure for cell number. Four days after treatment, a CellTiter-Glo® luminescent assay (Promega, catalog number G9242) was performed on the other 96-well plate to determine cell viability. The viabilities of DMSO-treated cells were set to 100%, and IC-50 curves were generated with the GraphPad Prism software. Experiments were repeated at least two to three times. To investigate the effect of *KAT2A* knockdown on NB cell proliferation, cells were transfected with *siCtrl* or *siKAT2A* and plated into 96-well plates, with three technical replicates for each condition. The experiments were performed twice in IMR32 and BE(2)C cell lines and once in KCNR cell line, serving as additional biological replicate. Statistical differences were assessed using a two-sided unpaired Student’s *t*-test.

### Protein isolation, western blotting analysis, and co-immunoprecipitation

To assess protein levels, cells were lysed using RIPA buffer, and 10 µg of total protein was separated by electrophoresis and electroblotted. The following primary antibodies were used: anti-MYCN antibody (Cat # sc-53993), anti-MAX antibody (Cat # sc-197), anti-KAT2B antibody (Cat # sc-13124), and anti-GAPDH antibody (Cat # sc-47724) from Santa Cruz Biotechnology, anti-H3K9ac (Cat # 91103) from Active Motif, anti-KAT2A antibody (Cat # ab217876) from Abcam; and anti-acetylated-lysine antibody (Cat# 9441) from Cell Signaling Technology. Protein bands were detected using a goat anti-rabbit (sc2004) or mouse IgG-HRP (sc2005) conjugated secondary antibody (200 μg/mL; Santa Cruz Biotechnology) and visualized with enhanced chemiluminescence (Amersham Biosciences).

To investigate protein-protein interactions, co-immunoprecipitation (co-IP) was performed as previously described with slight modification [36]. Parental IMR32 cells or HEK293T cells transiently transfected with FLAG-tagged KAT2A (FLAG-KAT2A) or HA-tagged MYCN (HA-MYCN) plasmid were lysed for 30 min in cold lysis buffer (50 mM pH 7.5 Tris-HCl, 137 mM NaCl, 1 mM DTT, 1 mM EDTA, 0.5% Triton X-100) supplemented with protease and phosphatase inhibitors (Halt protease and phosphatase inhibitor, Thermo) while shaking at 4 °C. MYCN antibody (Santa Cruz, sc-53993) (1 µg), normal IgG (1 µg), anti-HA-Tag mAb (Cell Signaling, Cat # 3724S) (2 µg), or Anti-FLAG M2 antibody (Sigma, Cat # F1804) (2 µg) was incubated with 50 µl Dynabeads M-280 sheep anti-mouse IgG magnetic beads (Thermo Fisher Scientific Cat# 11201D) or Dynabeads M-280 sheep anti-rabbit IgG magnetic beads (Thermo Fisher Scientific Cat# 11203D) in 200 µl wash buffer (50 mM pH 7.5 Tris-HCl, 137 mM NaCl, 1 mM EDTA, 0.5% Triton X-100) overnight at 4 °C with rotation. The clarified cell lysate (1–5 mg) was incubated with antibody-coupled magnetic beads in 1 ml of lysis buffer and agitated at 4 °C for 4 h. The beads were subsequently washed five times with wash buffer, and co-IP products were eluted by incubating with 25–40 µl 1x LDS-PAGE sample buffer supplemented with 10% β-mercaptoethanol, followed by boiling for 5 min. To validate the mass spectrometry result, co-IP and western blot were performed. For western blot analysis, the following primary antibodies were used: anti-MYCN (Santa Cruz Biotechnology, sc-5399), anti-MAX (Santa Cruz Biotechnology, sc-197), anti-KAT2A (Abcam, ab217876), anti-KAT2B (Santa Cruz sc-13124), anti-TADA2A (LSBio LS-C352901), and anti-TADA2B (Invitrogen PA5-57393). Protein bands were detected using a goat anti-rabbit or mouse IgG-HRP conjugated secondary antibody (200 μg/mL; Santa Cruz Biotechnology) or VeriBlot (Abcam ab131366) and visualized using enhanced chemiluminescence (Amersham Biosciences).

### Size exclusion

IMR32 cells were solubilized and extracted using the same lysis buffer and procedure as described above for the total protein preparation in the co-IP assay. The extracted proteins were separated by HPLC using a Sepax SRT SEC 300 column. The resulting fractions were analyzed by running them on a 4–20% SDS-PAGE gel, followed by western blotting to identify the fractions containing the indicated proteins.

### RNA-seq

Total RNA isolated from NB cells under various treatments (with three biological replicates) was subjected to RNA-seq analysis, as previously described [37]. RNA extraction was performed using the RNeasy Plus Mini Kit (Qiagen Inc.) following the manufacturer’s instructions. Strand-specific whole transcriptome sequencing libraries were prepared using either the TruSeq® Stranded Total RNA LT Library Prep Kit or the TruSeq Stranded mRNA Library Prep kit (Illumina, San Diego, CA, USA). Paired-end sequencing was carried out on an Illumina platform. The resulting Fastq files were processed using Partek Flow. Raw reads were aligned to the genome using STAR, and the aligned reads were quantified with Partek E/M to the annotation model. Normalization was performed using the counts per million (CPM) method in Partek Flow. Gene lists generated through Partek Flow were further analyzed using QIAGEN’s Ingenuity Pathway Analysis (IPA) and Gene set enrichment analysis (GSEA). By default, a false discovery rate (FDR) of less than 0.25 was considered significant for GSEA. GSEA uses permutation testing to estimate the significance of the enrichment score. When presenting RNA-seq data in bar graphs for representative genes, the results are shown as mean ± SE, based on three biological replicates. Statistical significance was determined using a two-sided unpaired Student’s *t*-test.

### Real-Time PCR

Total RNA extracted from NB cells was reverse-transcribed into cDNA. Quantitative measurements of β-ACTIN, and other gene expression levels were obtained using the BIO-RAD CFX Touch Real-Time PCR Detection System. Each measurement was performed in nine replicates, which included three biological regulates and three technical replicates per biological replicate. Ct values were normalized to β-ACTIN levels. Statistical differences were assessed using one-way ANOVA. KiCqStart primers (Millipore Sigma, Cat # KSPQ12012G) were used for the *ODC1, NOP58, TFRC*, and *TGIF1* genes. The primer sequences for β-ACTIN are as follows: Forward: GCCAACCGCGAGAAGATGA; Reverse: CATCACGATGCCAGTGGTA.

### ChIP-seq

ChIP-seq was carried out using the ChIP-IT High Sensitivity kit (Active Motif, cat. 53040) as described previously [37]. Cells were fixed with 1% formaldehyde for 13 min, and chromatin was sheared into 200–700 bp fragments using an Active Motif EpiShear Probe Sonicator. For IMR32 cells, sonication was performed at 25% amplitude, pulse for 20 s on and 30 s off for a total sonication “on” time of 16 min. The sheared chromatin was immunoprecipitated overnight at 4 °C with antibodies against MYCN (Active Motif, Cat # 61185) and KAT2A (Abcam, Cat # ab217876). ChIP experiments included Active Motif ChIP-seq spike in chromatin (Active Motif Cat No. 53083) and *Drosophila*-specific histone variant H2Ac (Active Motif Cat No. 61686). Libraries were prepared using the NEBNext Ultra II DNA Library Prep Kit (NEB, E7645) multiplexed, and sequenced on an Illumina NextSeq platform.

As described previously [37], ChIP-enriched DNA reads from home-generated ChIP-seq data were aligned to the human genome (version hg19) using BWA. The ChIP-seq read density was normalized to counts per million mapped reads. High-confidence ChIP-seq peaks were identified using MACS2 (https://github.com/taoliu/MACS) with the narrow peak option for each TF. Peaks were selected based on statistical significance thresholds (*p* < 10^−7^ for MYCN, *p* < 10^−5^ for KAT2A). The genomic distribution of these peaks (as intronic, intergenic, exonic, etc.) was annotated using HOMER. Enrichment of known and de novo motifs was determined using the HOMER script “find Motifs Genome.pl” (http://homer.salk.edu/homer/ngs/peakMotifs.html).

The peak sets were further analyzed using the deepTools2 suite (v3.3.0) [38]. Peak normalization to reads per kilobase per million reads (RPKM) was performed using the bamCoverage. The computeMatrix function was used to generate a matrix of signal intensity for proteins centered on their peaks (±500 bp, total 1000 bp) in 10 bp bins. The matrix was subsequently used to calculate the accumulated signal around each peak center, serving as the signal intensity for protein binding sites.

Metagene plots of signal intensity for ChIP samples were created using deepTools. Briefly, computeMatrix was used to calculate signal intensity scores for each ChIP sample within genome regions specified by a BED file. The resulting matrix file, containing scores from two ChIP samples, was used to generate heatmaps with the plotHeatmap function or metagene plots with the plotProfile function.

### Statistics and reproducibility

The sample sizes and statistical analyses used in this paper are detailed in the relevant figure legends and the Materials and Methods sections. For standard comparison, analyses were performed using standard two-tailed Student’s t-test or one-way ANOVA. For the correlation study, statistical significance was assessed using a two-tailed Pearson correlation coefficient analysis with a 95% confidence interval, conducted with the GraphPad Prism software.

## Supplementary information

Supplementary figures

Dataset 1

Dataset 2

Dataset 3

Dataset 4

Dataset 5

Dataset 6

## Data Availability

All the home-generated ChIP-seq and RNA-seq can be found in the Gene Expression Omnibus (GEO) database. GEO accession number for ChIP-seq data generated in this study is GSE269363, and for RNA-seq data is GSE26936. ChIP-seq data of histone marks in IMR32 and RNA-seq data of MYCN overexpression in SHEP cells can be found under GEO accession number GSE208424 [11]. RNA-seq of *MYCN* silencing for 72 h in IMR32 can be found under GEO accession number GSE183641 [33].
