# Supplementary material for: MYCN and KAT2A form a feedforward loop to drive an oncogenic transcriptional program in neuroblastoma
Source: Oncogenesis. 2025 Apr 24;14(1):13. doi: 10.1038/s41389-025-00557-2 (PMC12022051; doi:10.1038/s41389-025-00557-2)
Supplement: Supplementary file 1 — Supplementary figures [file 41389_2025_557_MOESM1_ESM.docx]

**MYCN and KAT2A form a feedforward loop to drive an oncogenic transcriptional program in neuroblastoma**

Zhihui Liu^1*^, Jason J. Hong^1^, Xiyuan Zhang^1^, Carly M. Sayers^1^, Wendy Fang^1^, Man Xu^1^, Sydney Loria^1^, Sakereh Maskal^1^, Haiyan Lei^1^, Haitao Wu^2^, Rolf Swenson^2^, Jordan L. Meier^3^, Jack F. Shern^1^, Carol J. Thiele^1*^

^1^Pediatric Oncology Branch, National Cancer Institute, Bethesda, MD, USA

^2^Chemistry and Synthesis Center, National Heart, Lung, and Blood Institute, Bethesda, MD, USA

^3^Chemical Biology Laboratory, National Cancer Institute, Frederick, MD, USA

^*^To whom correspondence should be addressed.

Zhihui Liu: National Cancer Institute; Building 10, CRC, Room 1W-3940; 10 Center Dr. MSC-1105; Bethesda, MD 20892; Tel: 1-240-858-3857; Fax: 1-301-451-7052; Email: [liuzhihu@mail.nih.gov](mailto:liuzhihu@mail.nih.gov).

Carol J. Thiele: National Cancer Institute; Building 10, CRC, Room 1W-3940; 10 Center Dr. MSC-1105; Bethesda, MD 20892; Tel: 1-240-858-3849; Fax: 301-451-7052; Email: [thielec@mail.nih.gov](mailto:thielec@mail.nih.gov).

Conflict of interest

The authors declare no conflict of interest.

**Legend of supplementary datasets**

**Supplementary Table 1.** Gene ontology analysis of MYCN nuclear protein partners identified in IMR32 cells.

**Supplementary Table 2.** Bed files generated by K-Means clustering of histone marks with MYCN and KAT2A ChIP-seq around MYCN binding sites (associated with Fig. 1G).

**Supplementary Table 3.** The knockdown of *MYCN* in IMR32 cells for 72 h reduced the KAT2A ChIP-seq peak number.

**Supplementary Table 4.** Gene expression changes after genetic silencing of *KAT2A* in IMR32 cells for 48 h.

**Supplementary Table 5.** Gene expression changes after GSK983 treatment of IMR32 cells for 24 h.

**Supplementary Table 6.** Comparison of the differentially expressed genes identified by genetic silencing of *KAT2A* and PROTAC-mediated KAT2A degradation through Venn diagram analysis.

**Supplementary figures**


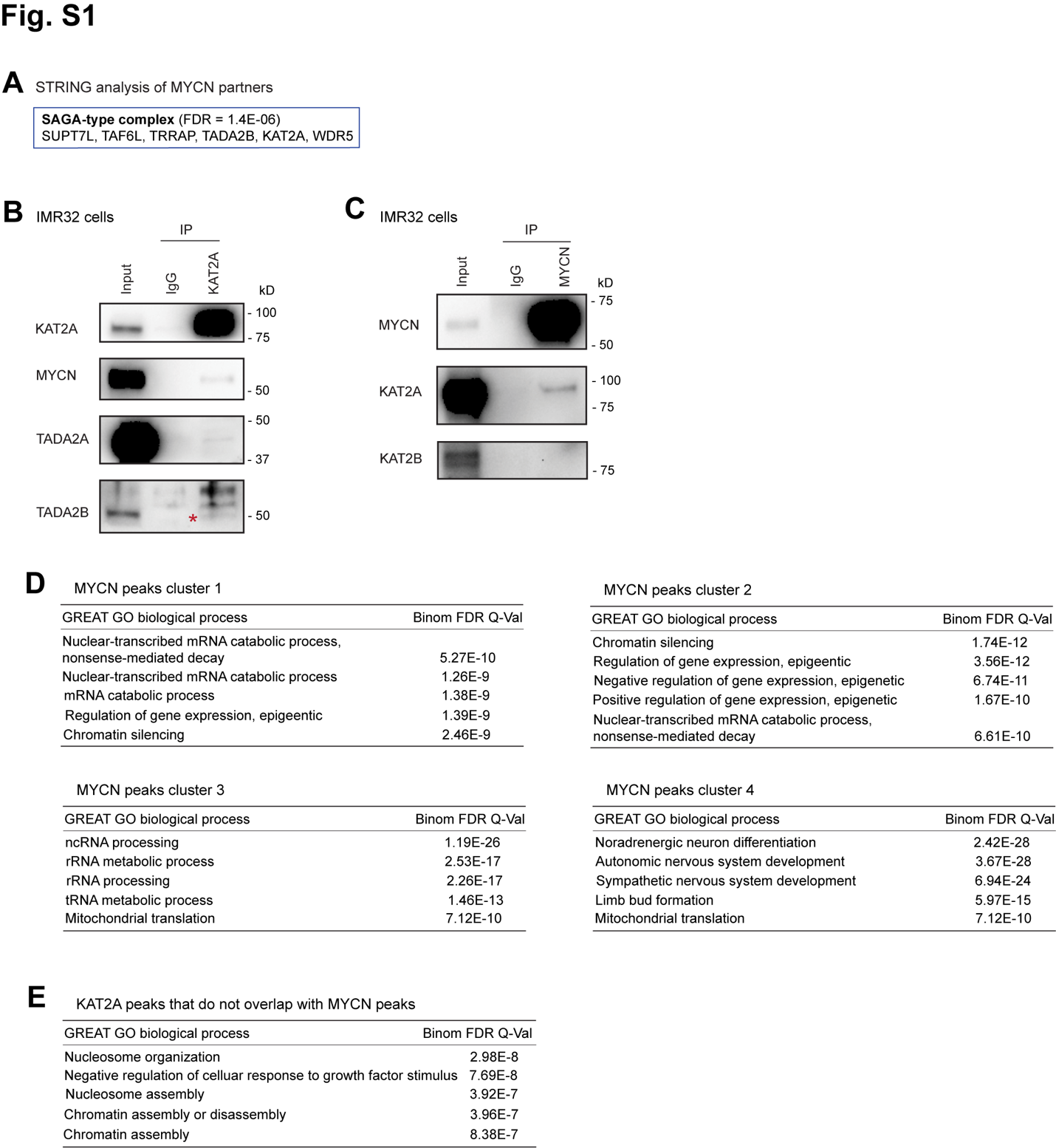


**Fig. S1. KAT2A is associated with MYCN (supplementary to Fig. 1). A.** STRING analysis of MYCN protein partners in the “chromatin organization” category shows a significant enrichment of the subunits of the SAGA-type complex. **B.** The IMR32 whole cell extracts are used for co-IP with anti-KAT2A antibody. Western blot assay shows the pulldown of KAT2A, MYCN, TADA2A, and TADA2B (Note: a faint band at the same molecular weight as TADA2B in the KAT2A co-IP lane was highlighted with a red asterisk). **C.** The IMR32 whole cell extracts are used for co-IP with anti-MYCN antibody. Western blot assay shows the pulldown of MYCN, KAT2A, but not KAT2B, even with extended enhanced chemiluminescence (ECL) exposure during western blot assay. **D.** GREAT GO analysis of four clusters of MYCN centered peaks indicated in Fig. 1G are associated with various biological processes. **E.** GREAT GO analysis of KAT2A unique peaks indicated in Fig. 1J that do not overlap with MYCN are associated with nucleosome and chromatin assembly.


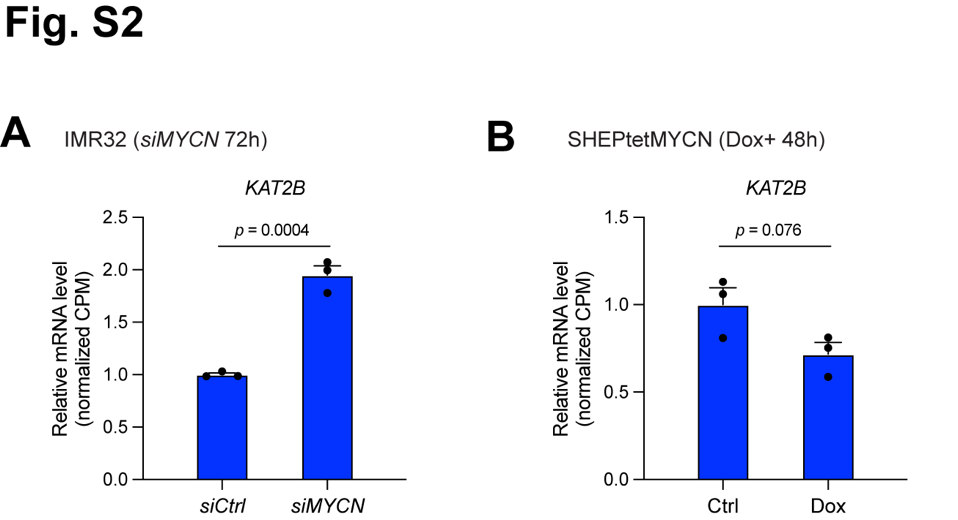


**Fig. S2. MYCN directly regulates KAT2A expression (supplementary to Fig. 2). A.** The effect of genetic silencing of *MYCN* in IMR32 cells on the mRNA expression levels of KAT2B was analyzed using the RNA-seq data from three biological replicates. Data are presented as mean ± SEM. CPM: counts per million. **B.** The effect of *MYCN* overexpression in SHEP cells on the mRNA expression levels of KAT2B was analyzed using the RNA-seq data from three biological replicates. Data are presented as mean ± SEM. CPM: counts per million.


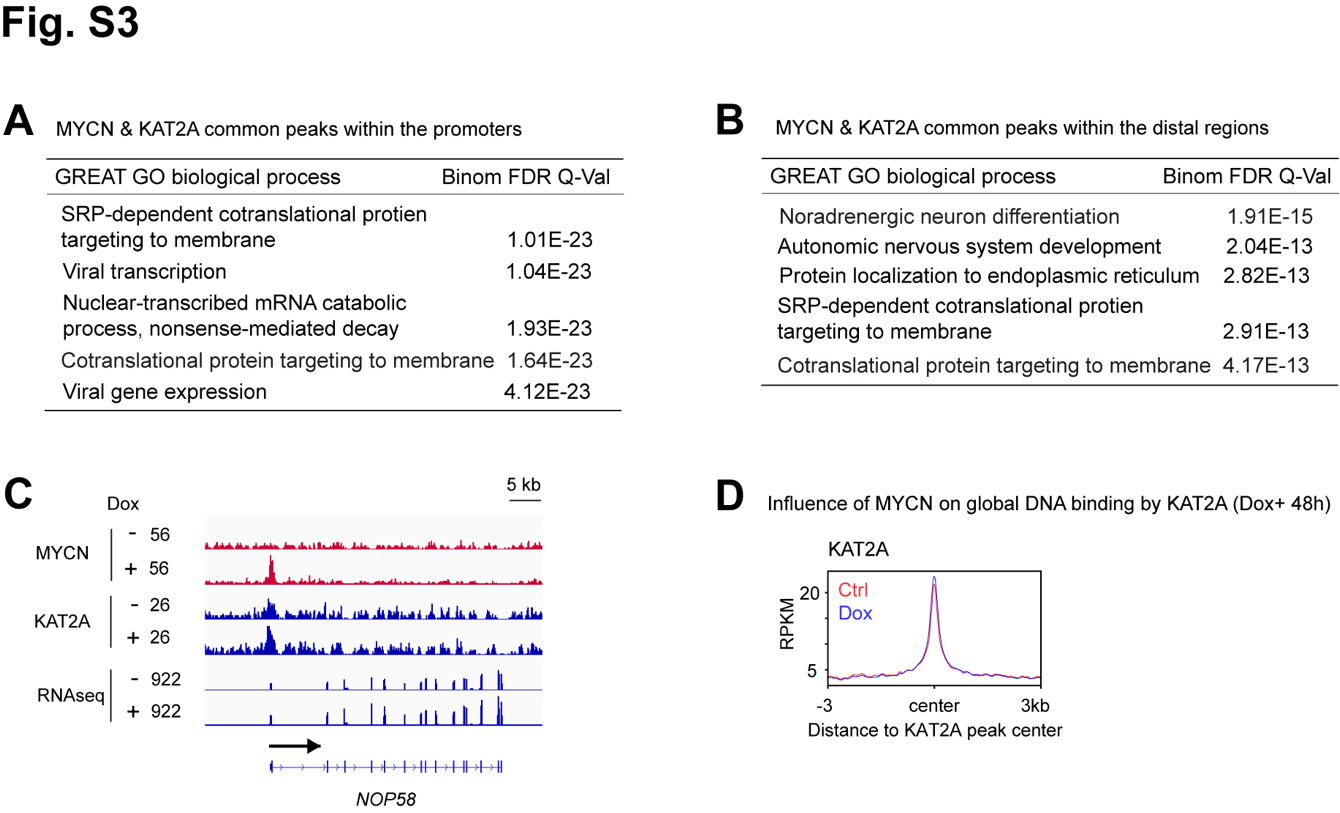


**Fig. S3. Silencing of *MYCN* alters genomic DNA binding of KAT2A (supplementary to Fig. 3). A.** GREAT GO analysis indicates that MYCN & KAT2A overlapped binding sites within the promoters are associated with protein translation and RNA processing. **B.** GREAT GO analysis indicates that MYCN & KAT2A overlapped binding sites outside of the promoters are associated with nervous system development. **C.** Signal tracks show that the overexpression of *MYCN* results in an increase in MYCN and KAT2A signals at the promoter of the *NOP58* gene. **D.** Metagene plots show the effect of *MYCN* overexpression in SHEP cells on KAT2A ChIP-seq signals when focusing on all KAT2A peaks.


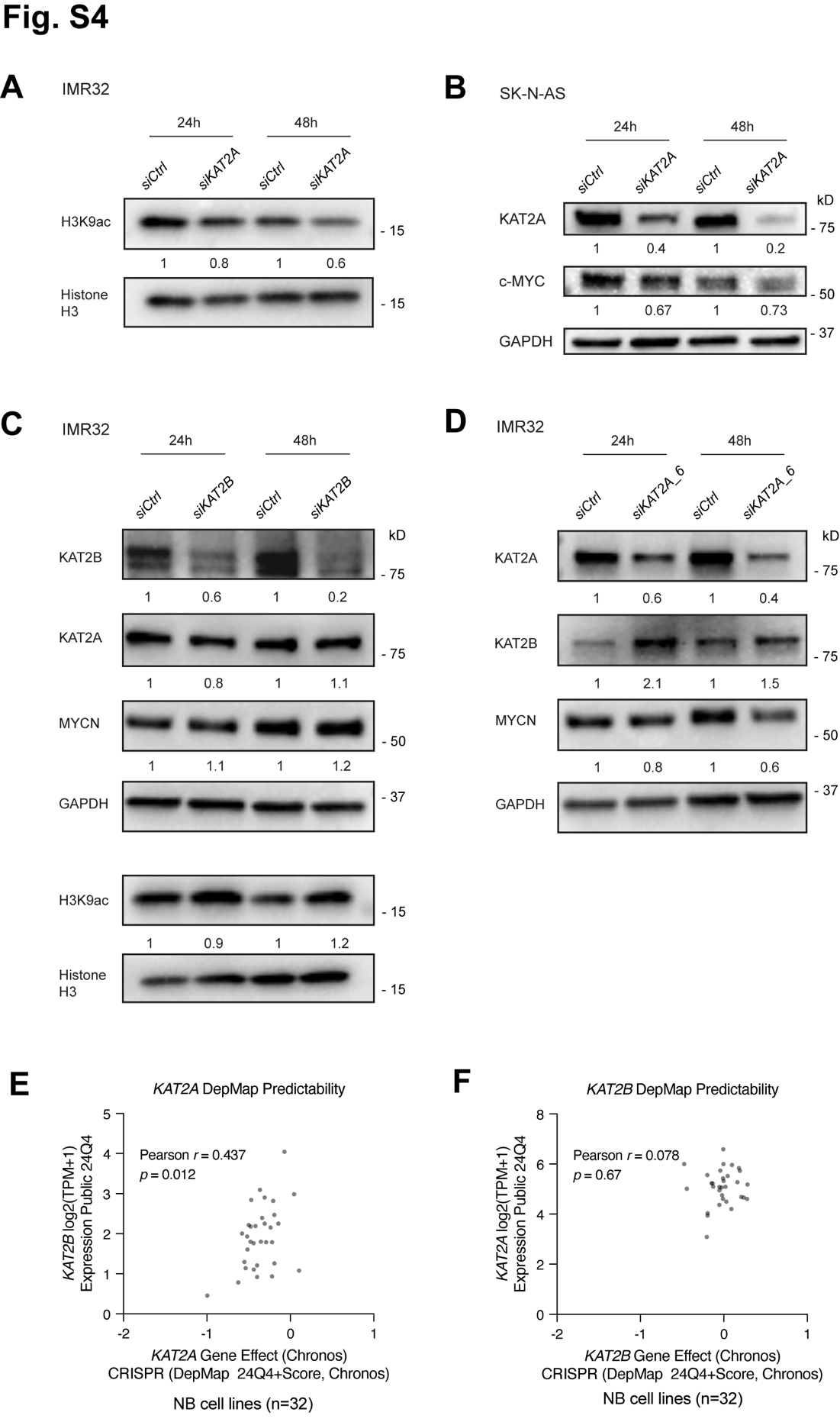


**Fig. S4. KAT2A regulates MYCN stability (supplementary to Fig. 4). A.** Knockdown of *KAT2A* in IMR32 cells results in a decrease in H3K9ac protein levels detected by western blotting assay. **B.** Knockdown of *KAT2A* in *MYCN* non-amplified NB cell line SK-N-AS results in a decrease in KAT2A and c-MYC protein levels detected by western blotting assay. **C.** Knockdown of *KAT2B* in IMR32 cells does not reduce KAT2A, MYCN, and H3K9ac protein levels detected by western blotting assay. **D.** Knockdown of *KAT2A* in IMR32 cells reduces MYCN protein levels and increases KAT2B protein levels detected by western blotting assay. **E.** The expression levels of *KAT2B* are significantly correlated with the CRISPR knockout effect of *KAT2A* in NB cell lines (Pearson r value = 0.437, p = 0.012). Note: Pearson correlation coefficient (r) value that is greater than 0.5 represents a strong positive, between 0.3 and 0.5 represents a moderate positive, and between 0 and 0.3 represents a weak positive correlation. **F.** The expression levels of *KAT2A* are not significantly correlated with the CRISPR knockout effect of *KAT2B* in NB cell lines (Pearson r value = 0.078, p = 0.67). Data information: In panels (**E** and **F**), statistical significance was assessed using a two-tailed Pearson correlation coefficients analysis with a 95% confidence interval.


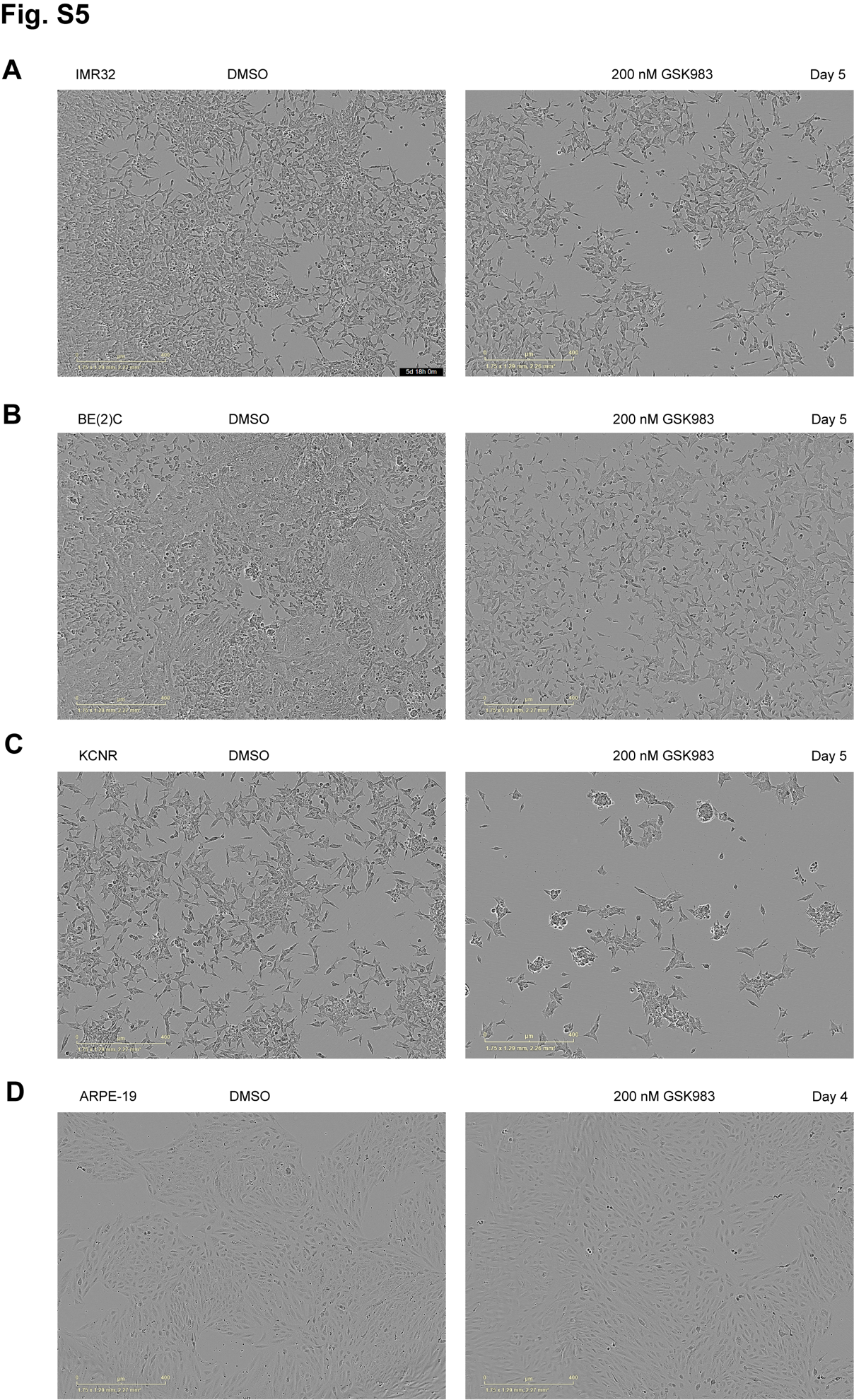


**Fig. S5. Targeting KAT2A with PROTAC degrader suppresses NB cell proliferation (supplementary to Fig. 6). A-D.** KAT2A PROTAC degrader GSK983 treatment of IMR32, BE(2)C, KCNR but not ARPE-19 cells leads to a reduction of cell numbers indicated by IncuCyte cell imaging.
